# Artificial Astrocytes Improve Neural Network Performance

**DOI:** 10.1371/journal.pone.0019109

**Published:** 2011-04-19

**Authors:** Ana B. Porto-Pazos, Noha Veiguela, Pablo Mesejo, Marta Navarrete, Alberto Alvarellos, Oscar Ibáñez, Alejandro Pazos, Alfonso Araque

**Affiliations:** 1 Departamento de Tecnologías de la Información y las Comunicaciones, Facultad de Informática, Universidad de A Coruña, Campus de Elviña, A Coruña, Spain; 2 Instituto Cajal, Consejo Superior de Investigaciones Científicas, Madrid, Spain; Centre national de la recherche scientifique, University of Bordeaux, France

## Abstract

Compelling evidence indicates the existence of bidirectional communication between astrocytes and neurons. Astrocytes, a type of glial cells classically considered to be passive supportive cells, have been recently demonstrated to be actively involved in the processing and regulation of synaptic information, suggesting that brain function arises from the activity of neuron-glia networks. However, the actual impact of astrocytes in neural network function is largely unknown and its application in artificial intelligence remains untested. We have investigated the consequences of including artificial astrocytes, which present the biologically defined properties involved in astrocyte-neuron communication, on artificial neural network performance. Using connectionist systems and evolutionary algorithms, we have compared the performance of artificial neural networks (NN) and artificial neuron-glia networks (NGN) to solve classification problems. We show that the degree of success of NGN is superior to NN. Analysis of performances of NN with different number of neurons or different architectures indicate that the effects of NGN cannot be accounted for an increased number of network elements, but rather they are specifically due to astrocytes. Furthermore, the relative efficacy of NGN vs. NN increases as the complexity of the network increases. These results indicate that artificial astrocytes improve neural network performance, and established the concept of Artificial Neuron-Glia Networks, which represents a novel concept in Artificial Intelligence with implications in computational science as well as in the understanding of brain function.

## Introduction

In Artificial Intelligence, connectionist systems are based on networks of interconnected artificial neurons that emulate brain neuronal networks [1], [2]. Astrocytes have recently emerged as cellular elements actively involved in the transfer and integration of information in the brain. Indeed, astrocytes receive, process and regulate synaptic information which had led to a new concept in neuroscience, i.e., that brain function results from the coordinated activity of astrocytes and neurons in neuron-glia networks [3]–[7]. However, the design of artificial neuron-glia networks, where astrocytes exchange information with neurons and which are endowed with similar properties of astrocyte-neuron communication in biological systems, is still lacking. Based on our current knowledge of nervous system function, such novel design seems a logical step to be followed by future artificial intelligence. We therefore designed artificial neuron-glia networks and investigated the consequences of the presence of artificial astrocytes on the performance of artificial neural networks.

## Results

### Artificial astrocytes improve neural network performance

We used multilayer feed-forward artificial neural networks with 3 to 5 layers (including input and output layers). We compared the performance efficiency to solve problems of artificial pure neural networks and the corresponding artificial neuron-glia networks, which included astrocytes that sensed and modulated neuronal connections. Artificial astrocytes were designed to resemble the signaling properties of biological astrocytes, which respond to neurotransmitters released under high synaptic activity [6], [8]–[11] and regulate neurotransmission in a larger temporal scale (i.e. hundreds of milliseconds and seconds) than fast neuronal and synaptic signaling (i.e. milliseconds) [6]. Consequently, artificial astrocytes 1) were stimulated by highly active neuronal connections, and 2) regulated neuronal connections with slow temporal time course. Hence, 1) astrocytes were stimulated when the associated neuronal connections were active for at least n out of m iterations (n: 2 to 3; m: 4, 6, 8), and 2) considering the time unit as a single iteration, astrocytic effects lasted 4 to 8 iterations, and the neuronal connection weights gradually increased (25%) or decreased (50%) if the associated astrocyte was active or inactive, respectively. Present neuron-glial networks had an artificial astrocyte for each neuron, and each astrocyte only responds to the activity of the associated neuron and modulates the connections of that neuron with neurons of the next (adjacent) layer. For simplicity, spatial spread of the astrocyte signal to other neurons or communication between astrocytes were not considered (see Discussion).

Artificial networks were challenged to solve four classification problems (obtained from the University of California Irvine Machine Learning Repository [12]) with different characteristics and complexities defined by the number of input variables and output parameters: 1) In Heart Disease (HD) problem, networks detected the presence or absence of disease analyzing 13 parameters from 303 patients (i.e., they were fed with 13 inputs and provided a single binomial output); 2) In Breast Cancer (BC) problem, they predicted the presence of cancer from 9 properties from 699 patients (i.e., 9 inputs; a binomial output); 3) In Iris Flower (IF) problem, networks classified 150 flowers displaying 4 characteristics (width and length of petals and sepals) into 3 different species (i.e., 4 inputs; 3 possible outputs); 4) In the Ionosphere (IS) problem, networks defined “good” or “bad” radar signals according to the state of the ionosphere analyzing 34 characteristics of 351 radar signals (i.e., 34 inputs; a binomial output).

NN were trained using genetic algorithms (GA) [13]–[15] and NGN were trained using a learning hybrid method combining GA and the neuron-glia algorithm (see Materials and Methods). We quantified the following parameters: 1) Training and Test accuracies: the accuracies reached during training and test; 2) Steady Training and Test accuracies: the training and test accuracies, respectively, reached at the end of the process (60, 210, 16 and 240 minutes for HD, BC, IF and IS problems, respectively); 3) Training and Test times: the mean time at which 95% of the respective steady accuracy was reached.

**Figure 1:**
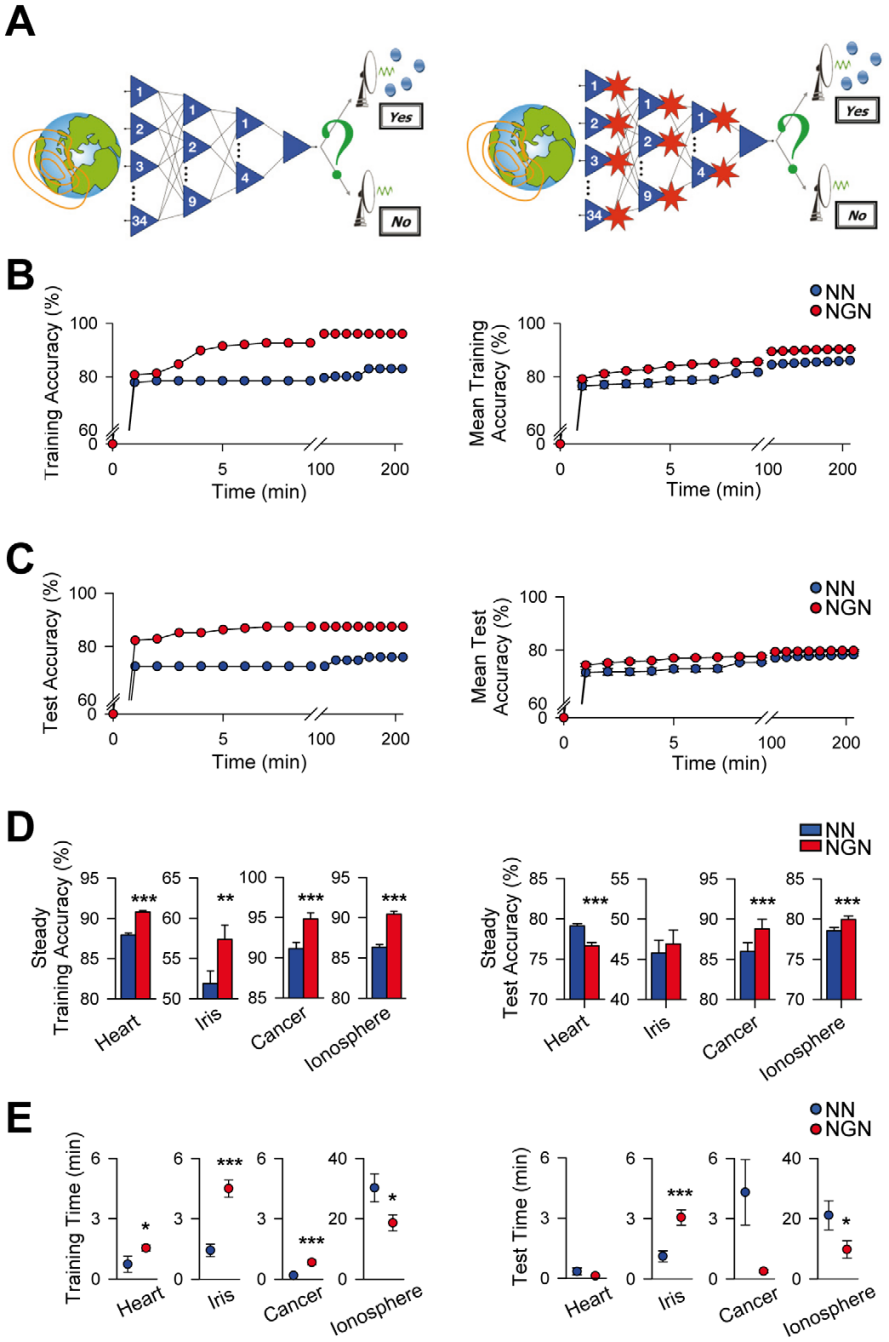
Artificial astrocytes enhance neural network performance. (**A**) Schematic drawing representing the design of artificial neural networks without (left) and with artificial astrocytes (red stars; right) designed to solve the Ionosphere (IS) problem. (**B**) Representative example (left) and mean training accuracy (n = 100) vs. time for the (NN) and (NGN) solving the IS problem. (**C**) Representative example (left) and mean test accuracy (n = 100) vs. time for the NN and NGN solving the IS problem. (**D**) Mean steady training and test accuracies (left and right, respectively; n = 100) of NN and NGN solving the four problems tested. (**E**) Mean training and test times (left and right, respectively; n = 100) of NN and NGN solving the four problems tested. *P<0.05, **P<0.01 and ***P<0.001. Values represent mean ± S.E.M.

When solving the IS problem (Figure 1A), both training and test accuracies of the NN increased over time until reaching a maximum at the end of the processes (Figure 1B, 1C). Similar behaviours were observed for the other problems. A similar developmental profile of both parameters over time was observed in NGN (Figure 1B, 1C). However, striking differences in the parameters were shown by NN and NGN.

The steady training accuracies of NGN were higher than the respective NN in all problems (Figure 1D). The steady test accuracy of NGN was also higher than NN when solving IS and BC problems, whereas it was reduced for HD problem, or unchanged for IF problem. Both training and test times of NGN and NN, yet in some cases significantly different, had similar values (<6 min) for HD, IF and BC problems (Figure 1E). In IS problem, which displayed long training and test times, both were shorter in NGN than in NN. These results indicate that astrocytes influenced the performance of the networks, without largely affecting or rather reducing their learning velocity. They also suggest that such influence depended on the network architecture and the problem tested.

### The improvement of network performance is specifically due to artificial astrocytes

**Figure 2:**
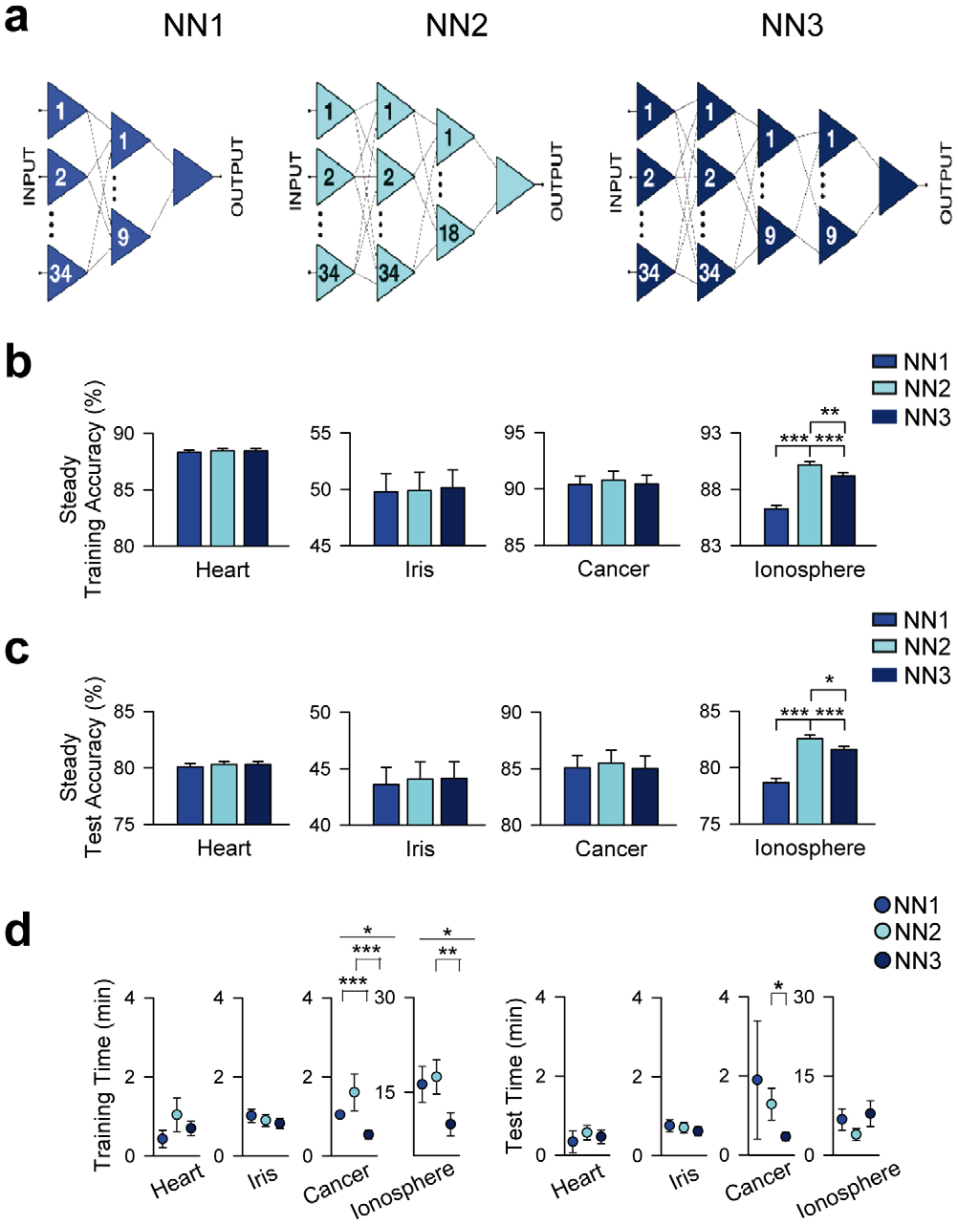
Neural network performance does not depend on the number of neurons or the architecture of the network. (**A**) Schematic drawing representing the design of three artificial neural networks with different number of neurons and different architectures. (**B and C**) Mean steady training and test accuracies, respectively (n = 100) of each NN for each problem tested. (**D**) Mean training and test times (left and right, respectively; n = 100) of each NN for each problem tested. *P<0.05, **P<0.01 and ***P<0.001. Values represent mean ± S.E.M.

Because the performance enhancement of NGN vs. NN might not be specifically due to astrocytes but to the presence of additional elements, we tested whether additional neurons in NN produced similar improvements. We analyzed the performances of NN with different architecture and number of neurons (Figure 2). We designed NN with 1, 2 or 3 hidden layers (NN1, NN2 and NN3) and with 44, 87 and 87 neurons (Figure 2A). In three problems (HD, IF and BC), no differences were found between the different NN (Figure 2B, 2C). In IS problem, accuracies were higher in NN2 and NN3 respect to NN1, but they were lower in NN3 than in NN2, which had the same number of neurons but different architectures, which is inconsistent with an improved performance as the number of neurons increase. Likewise, no trends were observed in training and test times (Figure 2D). These results indicate that NN performance did not correlate with the number of neurons or the architecture, suggesting that differences in NN and NGN performances cannot be accounted for an increased number of elements, but they are specifically due to astrocytes.

### Network performance improvement by artificial astrocytes increases as the network complexity increases

**Figure 3:**
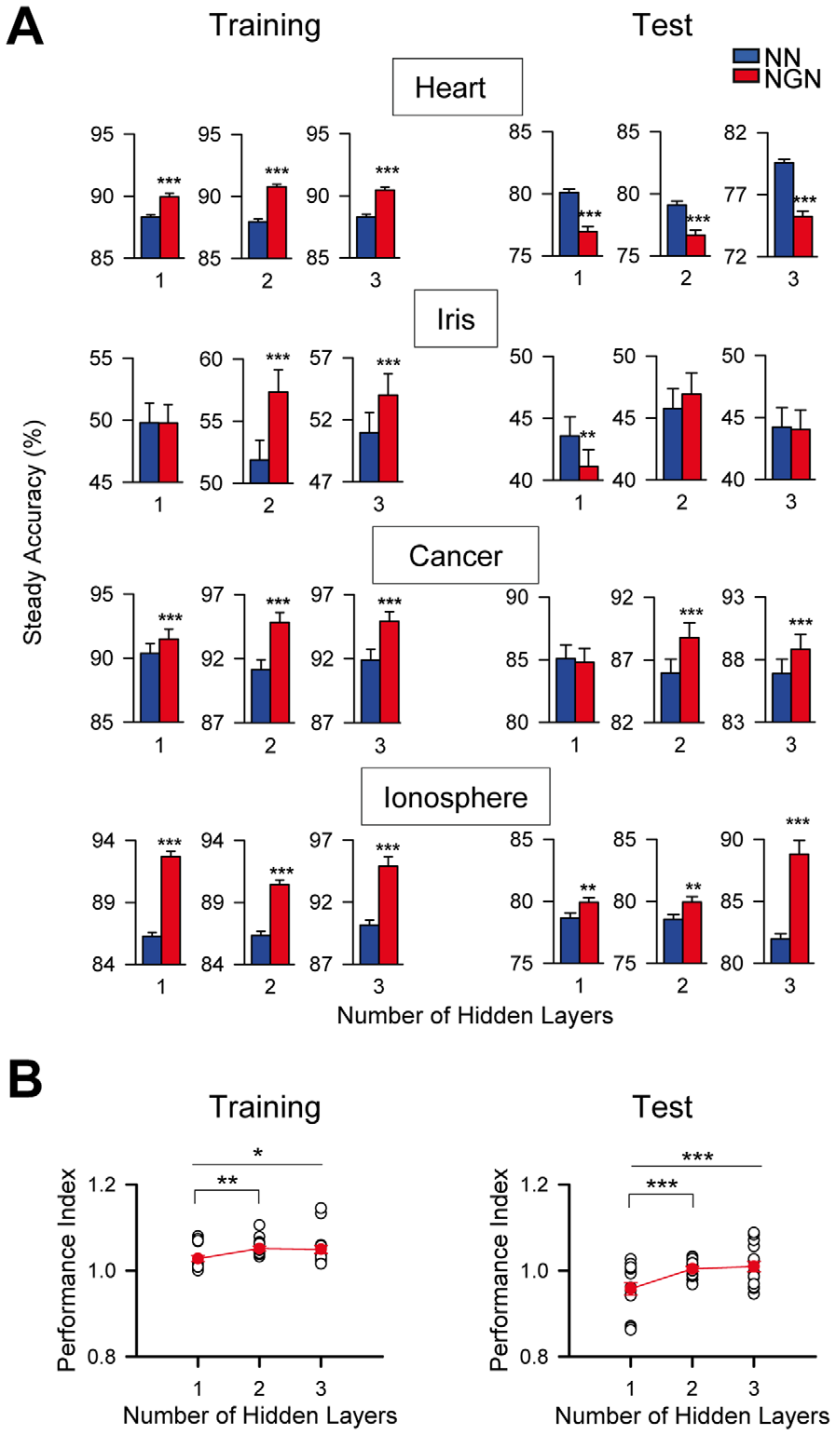
Network performance improvement by artificial astrocytes increases as the network complexity increases. (**A**) Mean steady training and test accuracies (left and right, respectively; n = 100) of NN and NGN with 1, 2 or 3 hidden layers to solve the four problems tested. (**B**) Performance indexes (i.e., NGN values relative to NN values) of the training and test accuracies (left and right, respectively). Red symbols represent the corresponding averaged values (n = 16). *P<0.05, **P<0.01 and ***P<0.001. Values represent mean ± S.E.M.

We next investigated whether astrocyte effects depended on the network complexity. We used networks with different levels of complexity (defined by their different number of neurons, hidden layers and connections) and compared their performances with the corresponding NGN. To quantify NGN vs. NN performance, we defined performance index as the ratio between steady accuracies of NGN and the corresponding NN. First, we analyzed the impact of astrocytes on three networks with different hidden layers for each problem tested (Figure 3A). The steady test and training accuracies of NGN and the corresponding NN were different, and their relative values were also different among the three networks (for each problem tested) (Figure 3A). Then, to estimate the astrocyte effects irrespective of the problem, we pooled together the performance indexes of the four problems and plotted vs. the number of hidden layers (Figure 3B). Both training and test performance indexes increased as the number of hidden layers increased (Figure 3B), indicating that the impact of astrocytes increased as the complexity of the network increased.

### Relative network performance improvement by artificial astrocytes depends on the problem tested

**Figure 4:**
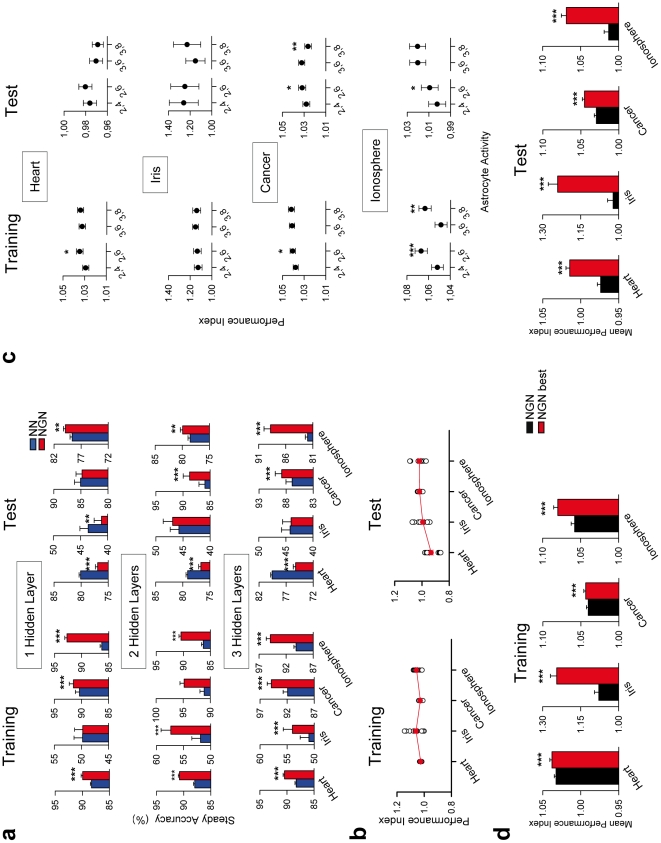
Relative network performance improvement by artificial astrocytes depends on the problem tested. (**A**) Mean steady training and test accuracies (left and right, respectively; n = 100) of NN and NGN with 1, 2 or 3 hidden layers to solve the four problems tested. (**B**) Performance indexes (i.e., NGN values relative to NN values) of the training and test accuracies (left and right, respectively). Red symbols represent the corresponding averaged values (n = 12). (**C**) Mean performance indexes of the training and test accuracies (left and right, respectively; n = 100) for each problem tested when artificial astrocytes were stimulated by different patterns of neuronal connection activity. The notation n,m indicates that artificial astrocytes were stimulated when the associated neuronal connections were active for at least n out of m iterations. (**D**) Mean performance indexes of the training and test accuracies (left and right, respectively; n = 100) for each problem of NGN with non-selected (black bars) or with specifically selected neuron-glia interaction parameters (red bars). *P<0.05, **P<0.01 and ***P<0.001. Values represent mean ± S.E.M.

We next asked whether astrocyte effects depended on the problem (Figure 4). In all cases (except IF problem, 1 hidden layer), the steady training accuracy and the performance index was increased in NGN vs. the respective NN, in all the problems and networks (Figure 4A, 4B). However, the steady test accuracy of NGN vs. NN displayed more variability depending on the problem (Figure 4A). To quantify the astrocyte impact irrespective of the network architecture, for each problem we pooled together the performance indexes of the three networks (Figure 4B). While the relative training accuracy was higher for IF and IS problems, the relative test accuracy increased following the sequence HD-IF-BC-IS (Figure 4B). This result indicates that the impact of astrocytes also depended on the problem tested.

### NGN performance improvement depends on intrinsic properties of astrocytes

Above results were obtained using a constant paradigm of astrocytic activation, i.e., astrocytes were stimulated when the associated neuronal connections were active for at least 3 out of 6 iterations. To investigate if NGN performance improvement depended on intrinsic properties of astrocytes, we analyzed whether different patterns of astrocytic activation influenced the performance indexes. We defined two variables in the artificial neuron-glia interaction: 1) Astrocytic Sensitivity as the number of times the neuronal connection was required to be active to stimulate the associated astrocyte, i.e., 2,m is more sensitive than 3,m (being m = 4, 6 or 8); 2) Neuron-glia Connection Power as the number of iterations in which the neuronal connections are possibly active to stimulate the astrocyte (for example, if n,m = 3,6, at least 3 activations of the neuron had to occur during 6 consecutive iterations to stimulate the associated astrocyte), consequently, the strength is: n,8>n,6>n,4 (being n = 2 or 3) because the ability of a neuron to stimulate the associated astrocyte is higher for m = 8 than m = 6 and m = 4. Figure 4C shows that the relative performance of NGN vs. the corresponding NN is variable depending on the sensitivity and the neuron-glia connection power, and is different for each problem, indicating that the relative improvement of NGN vs. NN depends on intrinsic properties of the astrocytes, i.e., their sensitivity to neuronal connection activity and the strength of the neuron-glia connection.

We finally investigated whether assigning specific values to the intrinsic properties of astrocytes and neuron-glia connections would further enhance the performance of NGN. We selected the best configuration of the neuron-glia interaction and compared it with the averaged non-selected configurations (Figure 4D). In all problems, the performance of the specifically designed NGN was enhanced vs. the corresponding NN (performance indexes >1) as well as vs. the corresponding NGN with non-selected configuration.

## Discussion

Present results show that the performance of artificial networks is improved by artificial astrocytes, which is in agreement and support recent experimental findings that propose a direct involvement of astrocytes in brain information processing [3]–[7]. The improvement provided by artificial astrocytes increases as the network complexity increases, which agrees with the gradual increase of the glia proportion observed in the phylogeny as the nervous system complexity increases [5], [16]. The specifically designed neuron-atrocyte properties provide a better network performance than indiscriminate properties, indicating that the interaction properties in these artificial tripartite synapses are relevant, which supports the notion that neuron-glia interaction in biological synapses represents a fine tuned communication [10].

Several mechanisms and physiological consequences of astrocyte-neuron communication occur [6], [17]. Under what conditions one specific modulatory effect takes place in a particular neural network remains unknown [17]. For simplicity and as a first approximation to a complex problem, present work focused in modelling astrocyte-induced synaptic potentiation to investigate whether artificial astrocytes improve artificial neural network performance. Once this proof of concept is established, the development of future models of astrocyte-neuron interaction that incorporate the richness of biological interactions, e.g., astrocyte-induced synaptic depression, or depression and potentiation altogether, as well as spatial spread of the astrocyte signalling and astrocyte-astrocyte communication, are required to test whether they provide similar, or even better, improvements of neural network performances. Likewise, future work is necessary to investigate the impact of astrocytes in more complex neural networks that include e.g., inhibitory neurons and/or feed-back neuronal communication.

In conclusion, the performance of artificial neural networks is improved when they include artificial astrocytes that are endowed with biologically-defined neuron-glia communication properties. Present results serve as foundation for the establishment of Artificial Neuron-Glia Networks, which represents a novel concept in Artificial Intelligence. Future developments of artificial neuron-glia networks will help to improve the efficacy of artificial networks as well as to better understand the role of astrocytes in brain function.

## Materials and Methods

### Architecture and Parameters

Table 1 shows the NN architectures used. In NGN, every astrocyte was associated with the neuronal connections of each neuron (i.e. HD, 1 hidden layer architecture, NN: 13-4-1 vs NGN: 13(13)*-4(4)*-1, where (n)* refers to n astrocytes).

**Table 1:** Architectures of NN used in each problem.

	One hidden layer	Two hidden layers		Three hidden layers	
	Fig. 2, 3, 4	Fig. 1, 3, 4	Fig. 2	Fig. 3, 4	Fig. 2
**Heart Disease**	13-4-1	13-4-3-1	13-13-8-1	13-5-4-3-1	13-13-4-4-1
**Iris Flower**	4-5-3	4-5-7-3	4-4-10-3	4-7-5-7-3	4-4-5-5-3
**Breast Cancer**	9-7-1	9-7-5-1	9-9-14-1	9-12-8-4-1	9-9-7-7-1
**Ionosphere**	34-9-1	34-9-4-1	34-34-18-1	34-12-8-4-1	34-34-9-9-1

The activation function was the hyperbolic tangent in all the layers, except in the output layer where the threshold function was used with a threshold value of 0.5 and an expected binary output.

The same initial population of individuals was used for each problem and architecture. The population sizes were 150 individuals (except for HD problem that was 100). The following techniques were employed: the Montecarlo method for the selection of individuals; the Darwinian substitution method; a single crossover point; a crossover rate of 90%; and a mutation rate of 10%.

The network architectures as well as GA parameters were selected for their simplicity [18] and to establish the same conditions for comparing NN and NGN.

### Hybrid learning method

We designed a new hybrid learning method for training the new NGN that searched for optimal connection weights in two phases. In one phase, the weight values were modified using rules based on neuron-glia communication properties [19]. In the other phase, the weights were adjusted through GA.

In the first learning phase, every individual (consisting of as many values as the connection weights exist in the NGN) of a population considered by the GA was modified as each training pattern passed on to the network, according to the activity of the neurons during the passage of that pattern. For each individual, every input pattern of the training set was presented to the network during m iterations (pattern cycle = m: 4, 6 or 8). These iterations modified the individual by applying an algorithm based on neuron-glia communication properties. This algorithm considered that the NGN had an artificial astrocyte for each neuron, and each neuron had an activity counter that begun with a value of zero and increased or decreased during each iteration in only one whole integer (+1 or −1) until it reached the Maximum (n) or Minimum (-n) Astrocytic Sensitivity. A neuronal connection ij connected neuron i with neuron j. A neuronal connection was considered active when the neuron i was active (according to its activation function). When the activity of a neuron reached its maximum value n, the astrocyte was activated and then increased 25% the weight of the neuronal connections with the neurons of the next (adjacent) layer. If a neuron that had reached this maximum value was once again activated, the value of n was maintained and the weights were increased another 25%. On the other hand, if the activity counter reached a value of –n, the astrocyte was not excited and the associated neuronal connection weights were decreased 50%. If a neuron had reached its minimum value and was not further activated, then the value of –n was maintained and the weights were further decreased. Therefore, the astrocytic effects were maintained and became gradually attenuated over time. The combinations (Astrocytic Sensitivity, Neuron-glia power connection: 2,4; 3,6; 2,6 y 3,8) were determined by trial-and-error, and allowed an upper limit of 3, 4, 5 or 6 astrocytic activations, respectively. Weight changes of 25% and 50% were chosen because they provided satisfactory results in the initial tests and they are in agreement with biological experimental observations, because being the increment lower than the decrement only neuronal connections with relatively high activity would remain reinforced [19].

Throughout the training phase, after pattern cycle finished the associated error was calculated. After all the training patterns were passed, the mean square error (MSE) for each individual was calculated. This phase constitutes a non-supervised training since the modifications of the connection weights did not consider the error of the output, but rather took place at any time according to the activation of astrocytes.

In the second learning phase, GA was applied to the individuals according to their MSE obtained in the first phase. The GA selected the new individuals with which the first and second phases were repeated until the pre-established stop-time was reached or no error was obtained.

During the test phase, the input patterns were presented to the network according to the combinations (Astrocytic Sensitivity, Neuron-glia power connection) determined in the training phase.

### Validation

For each problem and for each architecture, the values for the comparison of each NN with its corresponding NGN were the average precisions obtained in 100 different test results. These 100 tests were performed once each network was trained with 10 disjointed sets of input patterns using the 5 iterations of 2-fold crossvalidation method [20], and additionally employing ten different populations of initial weights. The sets of input patterns were divided evenly into 50% training and 50% testing patterns. Wilcoxon test [21] was used for statistics.

The steady test accuracies were measured after a training period that was previously established for each problem and architecture. This time was the same for NN and NGN and was the execution time associated with 5,000 generations of the 2,4 combination. Table 2 shows the stop times during the training phase.

**Table 2:** Stop times during the training phase (minutes).

	*One hidden layer*	*Two hidden layers*	*Three hidden layers*
**Heart Disease**	42	60	90
**Iris Flower**	7	16	26
**Breast Cancer**	180	210	360
**Ionosphere**	210	240	360

The simulations were performed with Linux operating system in the FINISTERRAE and SVG supercomputers from CESGA [22], Spain.
